# First Report of Hand, Foot, and Mouth Disease (HFMD) Outbreak in the West Bank, Palestine: Molecular Characterization of Coxsackievirus A16 (CV-A16)

**DOI:** 10.1155/cjid/9133821

**Published:** 2025-02-18

**Authors:** Kamal Dumaidi, Amer Al-Jawabreh, Areej Zraiqi, Athar Fashafsha, Ahmad Dumaidi

**Affiliations:** ^1^Department of Medical Laboratory Sciences, Arab American University Palestine, Jenin, State of Palestine; ^2^Leishmaniases Research Unit-Jericho, Jericho, State of Palestine; ^3^Faculty of Medicine, Arab American University Palestine, Jenin, State of Palestine

**Keywords:** CV-A16, enteroviruses, HFMD, Palestine, RT-nPCR

## Abstract

**Introduction:** Hand, foot, and mouth disease (HFMD) is a mild self-limited childhood infectious disease caused by a variety of enteroviruses (EVs).

**Aim:** To investigate the molecular epidemiology of EVs associated with HFMD and their clinical presentation during the HFMD outbreak that occurred in the Jenin district, Palestine, from May to August 2024.

**Materials and Methods:** Forty-four (44) throat and vesicular swabs were tested for enteroviral infections using two RT-PCR assays targeting both the 5′NTR and the VP1-2A regions of the enteroviral genome for the diagnosis and genotyping. Patients' demographic data and clinical history were used to create an epidemiological curve. EpiInfo free software was used to draw a cluster mapping. MEGA-X was used to construct a maximum likelihood (ML) tree. PopArt 1.7 software was used to construct neighbor-joining network.

**Results:** The mean age of the study sample was 2.08 (0.25–12 years) with 95% (42/44) under five years old. The male/female ratio was 0.9. All cases presented with typical HFMD signs and symptoms with variable sites of signs. Of the 44 samples, 36 yield positive RT-PCR targeting the 5′NTR. Seven randomly selected positive RT-PCR-5′NTR samples were sequenced using Sanger sequencing for genotyping. It was shown that all were CV-A16 sub-genogroup B1c. Phylogenetic analysis of VP1-2A region sequences showed that all Palestinian CV-A16 isolates form a pure haplogroup of CV-A16 sub-genotype B1c. Furthermore, although haplotype network analysis showed high variation between the viral sequences, the haplotype analysis supported the ML phylogenetic tree in having them all in one haplogroup.

**Conclusion:** CV-A16, sub-genotype B1c was the virus responsible for the HFMD outbreak in the Jenin district of Palestine in the summer of 2024. Phylogenetic and haplotype analysis showed that CV-A16 strains cluster closely with each other and very close to an Indian isolate (OR437338.1), indicating the monomorphic nature of this strain with low genetic variation and the probability of virus importation.

## 1. Introduction

Hand, foot, and mouth disease (HFMD) is a mild self-limiting illness caused by human enteroviruses (HEVs) of the *Picornaviridae* family. HEVs are nonenveloped, single-stranded, positive-sense RNA viruses that consist of approximately 7500 nucleotides (nt). The viral RNA genome contains a single open reading frame (ORF) flanked by nontranslated regions (NTRs) at each end. The ORF encodes a polyprotein that is post-translationally cleaved to yield four structural (VP4, VP2, VP3, and VP1) and seven nonstructural proteins (2A, 2B, 2C, 3A, 3B, 3C, and 3D). Enterovirus (EV) typing is based on comparing the sequences encoding the partial or the whole VP1 region of the viral genome: viruses of different genotypes have < 75% nucleotide identity and < 85% amino acid identity [1].

The HFMD may be acquired by direct contact with saliva, droplets, vesicular fluid, and fecal–oral route. The illness frequently affects children under 5 years old; however, adults may also rarely develop the disease [2]. Low-grade fever, blister-like rash on the hands, feet, knees, and buttocks, and sporadic ulcers caused by ruptured blisters inside the mouth are the disease's hallmarks [3]. However, severe neurological or cardiopulmonary sequelae have also been reported in severely infected children [4].

Enterovirus A71 (EV-71) and coxsackievirus A16 (CV-A16) have been found to be the predominant EV strains in the previous decades. Recently, coxsackievirus A6 (CV-A6) and coxsackievirus A10 (CV-A10) have been increasingly and have played key roles in a series of HFMD outbreaks, alongside a fluctuating increase in reported cases of CV-A16 every other year [5–10]. In addition, other EV strains belonging to HEV species B and C have also been reported from HFMD cases; however, the impact of these EV strains is still being investigated [11, 12]. Recently, an increase in the reported cases of HFMD with over 1.38 million cases per year in China, Taiwan, and other Asian countries has been noticed [7–10].

Currently, there are no standardized criteria for classifying the HFMD viral strains into sub-genotypes. In general, the CV-A16 strains are divided into 2 sub-genotypes: A and B, based on the entire or part of the VP1 sequence analysis. Sub-genotype B is further divided into sub-genotypes B1 and B2. Sub-genotype B1 can be further subdivided into sub-genotype B1a-1c. These sub-genotypes have been reported in China, Malaysia, Thailand, Australia, and Vietnam. This indicates that CV-A16 sub-genotypes co-evolve and co-circulate globally. Recently, new sub-genotypes C and D of CV-A16 have also been reported, further highlighting the dynamic nature of the virus [13–16].

In Palestine as well as in most of the surrounding countries in the region, including Jordan, Syria, Lebanon, and Egypt, there are no published reports about the epidemiology and genetic variation of EV serotypes that circulate and cause the HFMD. Therefore, the aims of the study were to investigate the molecular epidemiology of EVs associated with HFMD and their clinical presentation during the HFMD outbreak that occurred in the Jenin district, Palestine, from May to August 2024.

## 2. Materials and Methods

### 2.1. Study Area and Sampling

All HFMD-suspected patients were from the district of Jenin north of the West Bank of Palestine in the period from May 1 to August 1, 2024 (late spring and summer). The average temperature in the study area is 28°C in summer and 17.8°C in winter. The quantity of rainfall in 2024 was 593.7 mm with a relative humidity of 64% and a total evaporation rate of 1971 mm [17]. Jenin district has a population of 359,934 citizens, spread on an area of 583 km^2^ at density of 587 capita/km^2^. With Jenin city as the main city, the district contains 12 towns and more than 50 small villages and one refugee camp [18].

Patients' demographic data and the clinical history including the signs and symptoms were obtained by the treating physicians. Throat or blister swabs were collected from children seeking health care from private clinics using sterile swabs (Greiner Bio-One). The samples were collected during the acute phase of HFMD in the first week of illness with signs and symptoms of HFMD, including the appearance of vesicular and tender rash over, but not limited to, the palms of hand and soles of feet, with or without intraoral ulcers. The swabs were inoculated into 0.5 mL phosphate buffer saline (PBS) and transported to the Arab American University laboratory for viral investigations. All the samples were transported to the laboratory under cold chain conditions. The study received approval from the Ministry of Health in Palestine (reference number ATM/125/2024), which authorized the use of clinical samples and medical histories from anonymous children. Verbal consent, though undocumented, was obtained by the physician from the parents or guardians of the children.

### 2.2. RNA Extraction, Reverse-Transcriptase Nested Polymerase Chain Reactions (RT-nPCRs), and Sequence Analysis

Two hundred microliters from each clinical sample was used for viral RNA extraction using QIAamp Viral RNA/DNA mini extraction kit (Qiagen, Germany) according to the manufacturer's instructions. Five microliters of the final elution volume (45 μL) was used for EV diagnosis using RT-nPCRs targeting the 5′ NTR of the viral genome as described previously [19]. A band of 203 bp visualized on agarose gel electrophoresis indicated a positive result.

For EV genotyping by Sanger's sequencing, a second RT-nPCR protocol targeting 609 nts of the VP1-2A region of the EV viral genome was used as described previously [20].

The sequence analysis of the VP1-2A region of the seven randomly selected samples was used to identify the EV strains and sub-genotypes using the online Enterovirus Genotyping Tool (https://www.rivm.nl/mpf/enterovirus/typingtool) and/or a BLAST search of NCBI (National Center for Biotechnology Information; https://www.ncbi.ntlm.nih.gov/BLAST). The DNA sequencing data reported in this study have been submitted to GenBank under the accession numbers PQ381644, PQ381645, PQ381646, PQ381647, PQ381648, PQ381649, and PQ381650.

### 2.3. Statistical Analysis

Microsoft Excel (Microsoft Corporation, Redmond, Washington, United States) was used to create frequency table for the epidemiological (Epi) and clinical profile of HFMD outbreak cases. The analysis includes descriptive data using percentages and proportions. The Epi curve was constructed to show the distribution of cases over the period of the outbreak. Phylogenetic analysis was analyzed using MEGA-X software based on the 1000 bootstrap consensus maximum likelihood (ML) method [21]. A median-joining haplotype network was constructed using PopArt 1.7 based on single-nucleotide variation (SNV). Cluster mapping was created using EpiInfo free statistical package based on actual lat–long coordinates [22].

## 3. Results

### 3.1. Clinical Characteristics

Pediatricians and general practitioners (GPs) in the Jenin district of northern West Bank, Palestine, observed a rapid increase in mucocutaneous manifestations, including blisters on the tongue, buccal mucosa (lip and cheek lining), palms, and feet, suggestive of HFMD in children (personal communication). The number of HFMD cases continued to increase until August 2024, during which 44 random samples (9, 17, and 18 swabs from throat, hand, and foot, respectively) were collected with the assistance of the pediatricians and the GPs working in private clinics. The mean age was 2.08 years ranging from 3 months to 12 years. Apart from two cases, all HFMD cases were under five years old (42/44, 95%). The male/female ratio was 0.9. Fever (≥ 38°C) was reported in 13.6% (6/44) of the cases. All suspected cases presented with typical HFMD signs and symptoms with variable sites of symptoms as shown in Table 1. Neurological signs and symptoms including headache, irritability, coma, and drowsiness were not observed or experienced by the suspected patients.

### 3.2. Epi Distribution

**Figure 1:**
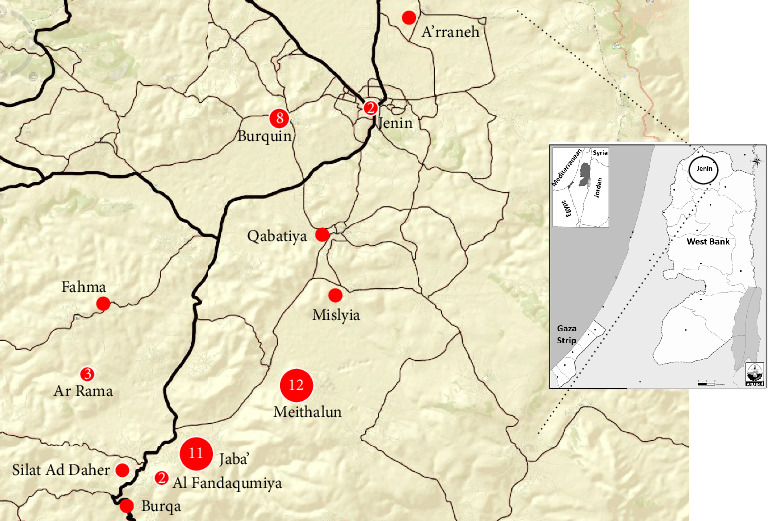
Cluster mapping of HFMD outbreak samples in Jenin district and the vicinity identified in the period from May to August, 2024.

**Figure 2:**
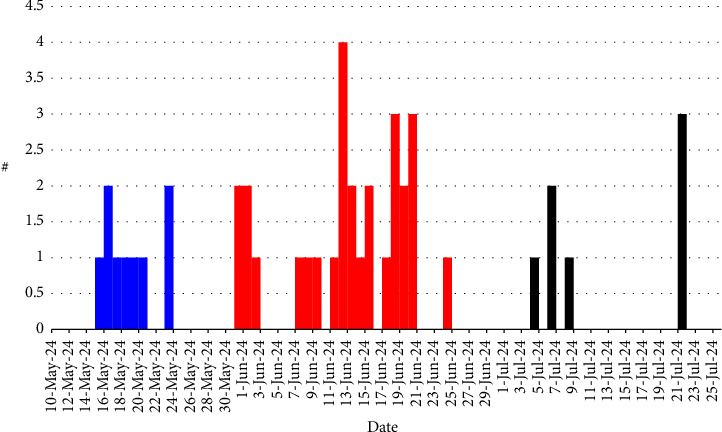
Epidemiological curve representing the periodic distribution of HFMD cases during the period between May and August 2024. The blue color represents May, the red color represents June, and the black color represents July.

The person–place–time model was used to describe the behavior of HFMD as a self-limited disease that occurs in the form of outbreaks. The HFMD outbreak in the district of Jenin was restricted to children less than 12 years old and equally distributed between sexes (Table 1). This HFMD outbreak was restricted to the city Jenin and eleven towns and villages in the vicinity (Figure 1). Two of these localities (Meithalun and Jaba' towns) contained more than half of the cases (52%). In addition, the first cases were reported in May in these two towns and outbreak ended in one of the two towns, Meithalun town. The epidemic curve (Figure 2) as a bar graph of cases versus time revealed that cases were reported in May, June, and July with June as the peak month. HFMD cases were spread over the outbreak time and merely confined to any given point of time. As shown in Figure 2, the spread of cases prolonged over the three-month outbreak. Given that the HFMD incubation period ranges from 3 to 6 days, the outbreak pattern aligns with a continuous exposure pattern rather than being confined to a single point in time. The pattern of this outbreak does not fit with other known patterns such as the classic epidemic curve (single common source exposure) or the multiple peaks seen in intermittent pattern.

### 3.3. Molecular Diagnosis

All 44 samples were tested using pan-EV RNA by 5′ NCR RT-PCR in primary screening. Among the tested samples, 36 samples from 44 cases (81.8%) were positive in the primary screening. For genotyping purposes, seven randomly selected positive 5′ NCR RT-PCR samples were used to amplify the part VP1-2A region of the viral genome using RT-nPCR and were sequenced using Sanger sequencing for genotyping (Table 1). All the seven randomly sequenced samples were found to be CV-A16, sub-genogroup B1c.

### 3.4. Genetic Clustering

**Figure 3:**
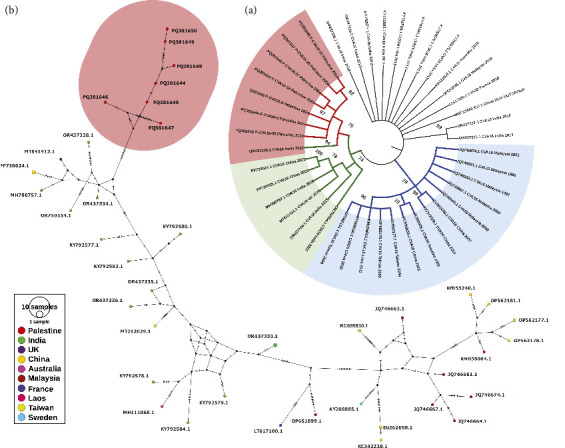
(a) A VP1-2A consensus maximum likelihood (ML) phylogenetic tree of the seven randomly sequenced HFMD cases along with 35 randomly GenBank-retrieved HFMD sequences. Bootstrap percentages above 70% are shown. An outgroup sequence (MW713345) was included. The three colors, red, green, and blue, are encompassing the three major clusters. (b) A haplotype median-joining network of the same sequences. Node colors represent countries of origin of sequences, as indicated in the color map key. Each node represents a haplotype. The diameter of the node circle is proportional to the number of sequences. The number of hash marks on the connecting lines between nodes indicates the number of mutations between nodes. The small black circles indicate inferred alleles due to missing sequences.

The ML phylogenetic tree of the 42 CV-A16 partial VP1-2A sequences retrieved from GenBank (Table 1 Supporting Information), including the seven sequences reported in this study, showed three major distinct clusters (Figure 3(a)). The first cluster includes the red clade (*n* = 8) which is exclusively Palestinian with one sequence from India which was closer to the Palestinian sequences than to its counter parts from India. The green clade (*n* = 6), adjacent to the Palestinian one (red), consisted mainly of Indian and Chinese sequences. The second cluster (blue) which is the largest (*n* = 13) of all consisted of Southeast Asian and Chinese sequences and one European (Swedish) sequence. The third cluster (black), which included the rest of the sequences (*n* = 13), did not show any clear clustering, but rather sporadic individual sequences. The haplotype (Figure 3(b)) network showed 40 haplotypes (colored nodes or circles) representing 40 alleles out of 42 sequences (haplotype: number (*h*: *n*) ratio = 0.95) reflecting high intraspecific variation between sequences. None of the haplotypes (nodes) contained multiple sequences, and thus circle sizes remained equally small with haplotype network spread over relatively large area. The hash marks between nodes represent the number of mutations or nucleotide differences between sequences. Lines crossed by three or more hash marks indicate 32 mutations. With regard to the Palestinian sequences (*n* = 7), the haplotype network supported the ML phylogenetic tree in having them all in one pure haplogroup. Next to the Palestinian haplogroup but with many nucleotide differences (hash marks) lies the mainly Indian haplogroup. The Southeast Asian/Chinese genogroup (blue) represented in the ML tree found its exact reflection in the right terminal end of the haplotype network. The haplotype network contained 47 small black circles with the polygons they formed indicating inferred alleles absent from datasets. The haplotype network showed sporadically distributed haplotypes with many inferred alleles in between the Indian and Southeast Asian/Chinese haplogroups which is in full congruence with the ML phylogenetic tree.

## 4. Discussion

So far, this is the first report on HFMD in Palestine as it is not considered a reportable disease. HFMD is considered a public health problem of global concern especially in several Asian countries [23]. Although it is a self-limiting disease, HFMD may develop into severe neurological and cardiopulmonary complications that could be fatal with EV-specific strains [24–26]. Therefore, investigating the sporadic and outbreak cases of HFMD is essential in monitoring the severe cases and the EV strains and variants, especially EV-71, CV-A16, and other EV strains that may lead to severe or deadly HFMD complications [27].

All HFMD study cases in the present study were mild. The signs and symptoms characterized by the typical clinical features of HFMD including the distributions and morphologies of mucocutaneous manifestations such as blisters were consistent with several previous studies. However, some studies reported that some EV strains and genogroups may be responsible for severe and even increased mortality rate among infected children [10, 23, 25, 28]. Fortunately, no severe HFMD complications including pulmonary edema and respiratory failure due to encephalitis and myocarditis were reported in this study as described by others [10, 23, 25]. The differences in the severity of the HFMD signs and symptoms among different studies may reflect differences in the virulence of the circulating viral strains and in population demographics.

The mean age of infected children in the HFMD outbreak was 2.08 years (3 months–12 years) which is in full agreement with other studies [10, 29, 30]. In general, the immune systems of infants and young children, which have not been exposed to infectious microbes and agents—including the viruses that cause HFMD—are more susceptible to contracting the disease due to their naïve and inadequately exercised immune systems. Also, inadequate cleaning and disinfection of toys, tableware, tables, chairs, and floors and bad hygiene in kindergarten and schools in general, especially hand hygiene among children, will enhance the propagation and transmission of pathogens [23, 31]. Furthermore, the vast majority of the HFMD cases in this study occurred in rural area which was shown to be a risk factor [3].

Although most studies have shown tendency of HFMD toward males, our study showed that HFMD is insignificantly higher in females (male:female = 0.9) which came parallel to couple of studies elsewhere [25, 31]. This could be partially explained by the type of sampling method which relied mainly on purposefully selecting the patients seeking health care.

Similar to those usually reported in Europe or Asia which are characterized by a sharp rise in the number of HFMD cases in summer and early autumn [32–34], the HFMD cases of the investigated outbreak were reported from May to July, 2024, with a sharp rise in June. Tang et al. reported that the HFMD outbreaks appear later in time as latitude goes north of the equator [35]. Nevertheless, other factors like climate were shown to affect the peak of the HFMD outbreaks. In China, peaks in warmer geography in the southern provinces with lower latitude occurred in spring and autumn while those in relatively colder ones in the north with higher latitude occurred in summer [3]. The northern West bank especially Jenin district, Palestine, is located at a low altitude of 32.4546°-North which aligns with the level of southern province of Jiangsu 32.0584°-North, with both having the same HFMD peak in late spring and summer.

The EV genotype in our HFMD outbreak based on the sequences of the VP1-2A region of the viral genome was identified as CV-A16 in 7/7 (100%). Decades earlier, both the EV-71 and CV-A16 were the most reported EV strains among HFMD cases globally [10, 36, 37]. However, the proportion of EV-71 among HFMD sporadic or outbreak cases decreased significantly especially after the launch of EV-71 vaccine in China in 2016. Recently, several reports showed that CV-A6 and CV-A10 are gradually replacing EV-71 and CV-A16 [37–39]. Nevertheless, Hu et al. showed that the prevalence of CV-A16 from HFMD cases had a fluctuating pattern in Beijing, China, from 2010 to 2018, increasing every other year [13]. Also, Guo et al. showed in their 12-year surveillance study that CV-A16 remains prevalent among HFMD cases [40]. CV-A16 has two sub-genotypes: sub-genotypes A and B. Sub-genotype B is divided into sub-genotypes B1 and B2, which are further divided into sub-genotypes B1a, B1b, and B1c and B2a, B2b, and B2c, respectively, with the predominance of sub-genotype B1 among recently reported HFMD outbreaks [41]. CV-A16 isolates in the present study belonged to the sub-genotype B1c. The sub-genotype B1c was reported recently in India and China from HFMD outbreaks [42–44].

Phylogenetic analysis of partial VP1-2A gene sequences revealed that all Palestinian CV-A16 positive cases form a single clade with an Indian isolate (OR437338.1), clustering with other CV-A16 strains, primarily from India and China, within the B1c clade. Additionally, haplotype network analysis showed a higher haplotype-to-sequence ratio (*h*: *n*), absence of large circles, increased hash marks, and a spread haplotype, indicating high variation between the viral sequences. However, this was consistent with the ML phylogenetic tree, which placed all the sequences in one distinct haplogroup. These findings suggest that the Palestinian sequences likely emerged from Indian haplotypes, but with significant genetic distance, as represented by the large number of hash marks. Furthermore, the phylogenetic results support previous studies indicating a recent increase in the reporting of CV-A16, clade B1c, in HFMD outbreaks [44, 45].

Despite including most of the sequences available in the GenBank, one limitation of the study is the need for more sequences to minimize the inferred alleles and thus provide a truer picture of the genetic variation within the HFMD viral population. The study samples were restricted to those collected from private clinics as the disease is not considered as a notifiable disease by the health authorities in Palestine due to its self-limited nature which might not have reflected the real picture of the outbreak.

In conclusion, CV-A16, sub-genotype B1c, was identified as the virus responsible for the HFMD outbreak in the Jenin district during the summer of 2024. Phylogenetic and haplotype analysis showed that the CV-A16 strains detected in the present study cluster closely with each other and very close to an Indian CV-A16 strain (OR437338.1), indicating the monomorphic nature of this viral strain with low genetic variation and the probability of virus importation. This study paves the way for the initiation of HFMD screening program in Palestine to deeply comprehend the epidemiology and behavior of the disease.

## Figures and Tables

**Table 1:** Epidemiological and clinical characteristics of HFMD-suspected and laboratory-confirmed cases.

Parameter	HFMD-suspected cases
Total number	44 cases
Age means (range)	2.08 (3 mon–12 years)
Male	21
Female	23
Male/female ratio	0.9
Vesicles	
Mouth	44 (100%)
Hand	42 (95%)
Foot	35 (80%)
Diarrhea	10 (22.7%)
Skin rash	1 (2.2%)
Month of sample collection-*N* (%)	
May-2024	11 (25%)
June-2024	27 (62.4%)
July-2024	6 (13.6%)
Sample type-*N* (%)	
Throat swab	9 (20.45%)
Hand vesicle swab	17 (39%)
Foot vesicle swab	18 (40.9%)
RT-nPCR (5′ NCR) positive	36/44 (81.8%)

## Data Availability

The data used in this study are available from the corresponding author upon reasonable request.

## Supporting Information

Table 1: Supporting: The CV-A16 partial VP1-2A sequences retrieved from GenBank.
