# Supplementary material for: First Report of Hand, Foot, and Mouth Disease (HFMD) Outbreak in the West Bank, Palestine: Molecular Characterization of Coxsackievirus A16 (CV-A16)
Source: Can J Infect Dis Med Microbiol. 2025 Feb 18;2025:9133821. doi: 10.1155/cjid/9133821 (PMC11858712; doi:10.1155/cjid/9133821)
Supplement: Supporting Information — Additional supporting information can be found online in the Supporting Information section. [file 9133821.f1.pdf]

Table 1. Supplement: The CV-A16 partial VP1-2A sequences retrieved from GenBank

#	**Accession no.**	**Country**	**Date of detection**	**Type**
1	OR437334.1	India	2013	CV-A16
2	MT641412.1	UK	2018	CV-A16
3	MH780757.1	India	2018	CV-A16
4	OR437338.1	India	2022	CV-A16
5	PP730024.1	China	2022	CV-A16/B1c
6	PP730025.1	China	2022	CV-A16/B1c
7	KY792583.1	India	2013	CV-A16
8	OR750554.1	India	2022	CV-A16
9	KY792580.1	India	2013	CV-A16
10	OR437335.1	India	2013	CV-A16
11	OR437336.1	India	2018	CV-A16
12	KY792577.1	India	2012	CV-A16
13	KY792584.1	India	2015	CV-A16
14	MT212029.1	China	2017	CV-A16
15	MH111068.1	Australia	2017	CV-A16
16	OR437333.1	India	2013	CV-A16
17	OR437337.1	India	2018	CV-A16
18	KY792578.1	India	2013	CV-A16
19	KY792579.1	India	2013	CV-A16
20	JQ746661.1	Malaysia	1999	CV-A16
21	JQ746664.1	Malaysia	2000	CV-A16
22	JQ746663.1	Malaysia	1997	CV-A16
23	JQ746667.1	Malaysia	2000	CV-A16
24	LT617100.1	France	2010	CV-A16/B
25	KM055004.1	France	2011	CV-A16/Ba
26	KC342228.1	China	2012	CV-A16
27	OP562181.1	Taiwan	2008	CV-A16/B1
28	KC695830.1	China	2013	CV-A16
29	JQ746674.1	Malaysia	2002	CV-A16
30	AY208085.1	Swedin	2002	CV-A16
31	KF055240.1	China	2010	CV-A16
32	OP562178.1	Taiwan	2005	CV-A16/B
33	EU262658.1	China	2007	CV-A16
34	OP562177.1	Taiwan	2004	CV-A16/B1
35	OP651899.1	Malaysia	2018	CV-A16
36	MW713445.1	China	2018	Ev-71 (outgroup)
